# Optimising cervical cancer screening during pregnancy: a study of liquid-based cytology and HPV DNA co-test

**DOI:** 10.1017/S095026882400013X

**Published:** 2024-01-23

**Authors:** Liying Gu, Yuan Hu, Yingting Wei, Zubei Hong, Yu Zhang, Jianhua Lin, Lihua Qiu, Wen Di

**Affiliations:** 1 Department of Obstetrics and Gynecology, Ren Ji Hospital, Shanghai Jiao Tong University School of Medicine, Shanghai, China; 2 Shanghai Key Laboratory of Gynecologic Oncology, Ren Ji Hospital, Shanghai Jiao Tong University School of Medicine, Shanghai, China; 3 State Key Laboratory of Oncogenes and Related Genes, Shanghai Cancer Institute, Ren Ji Hospital, Shanghai Jiao Tong University School of Medicine, Shanghai, China

**Keywords:** cervical cancer, cervical intraepithelial neoplasia (CIN), HPV infection, pregnancy, TCT

## Abstract

This study assessed the efficacy of ThinPrep cytologic test and human papillomavirus (HPV) co-test in cervical cancer screening during pregnancy. A cohort of 8,712 pregnant women from Ren Ji Hospital participated in the study. Among them, 601 (6.90%) tested positive for high-risk HPV (HR-HPV) and 38 (0.44%) exhibited abnormal cytology results (ASCUS+). Following positive HR-HPV findings, 423 patients underwent colposcopy, and 114 individuals suspected of having high-grade squamous intraepithelial lesion and cervical cancer (HSIL+) underwent cervical biopsy. Histological examination revealed 60 cases of normal pathology (52.63%), 35 cases of low‐grade squamous intraepithelial lesion (30.70%), 17 cases of HSIL (14.91%), and 2 cases of cervical cancer (1.75%). The incidence of HSIL+ in HPV 16/18 group was significantly higher than that in non-HPV16/18 group (10.53% vs. 6.14%, *P* < 0.05). Subsequent evaluation of the clinical performance of cytology alone, primary HPV screening, and co-testing for HSIL+ detection revealed that the HSIL+ detection rate was lowest with cytology alone. These findings suggest that HPV testing, either alone or combined with cytology, presents an efficient screening strategy for pregnant women, underscoring the potential for improved sensitivity in cervical cancer screening during pregnancy. The significantly higher incidence of HSIL+ in the HPV16/18 group emphasizes the importance of genotype-specific considerations.

## Introduction

Cervical cancer remains a prevalent malignancy affecting women globally, ranking as the fourth most common cancer. In 2020, the World Health Organization reported an estimated 604,127 new cases and 341,831 deaths attributed to cervical cancer worldwide [1]. Notably, within China’s female reproductive health landscape, the significance of this disease is underscored by approximately 99,000 new cases annually, constituting 18% of the global incidence, with around 30,000 associated deaths [2]. The latest data from the 2019 national cancer report by the National Cancer Center underscores the prominence of cervical cancer, ranking it among the top 10 malignancies and as the sixth most frequent cancer in females. Furthermore, cervical cancer holds the foremost position among tumours affecting the female reproductive system [3–4]. The human papillomavirus (HPV), a sexually transmitted double-stranded DNA virus, includes over 200 genotypes, with more than 50 affecting the reproductive tract and anal mucosa. The current understanding emphasizes that persistent infection with high-risk HPV (HR-HPV) is a requisite factor for the development of cervical precancerous lesions or cervical cancer [5]. In addressing the limitations of cytological screening, which is characterized by substantial subjective factors and diminished sensitivity [6], HPV typing has emerged as a crucial tool for cervical cancer screening [7]. This method effectively complements and enhances existing screening strategies, offering a more objective and sensitive approach to the detection of HPV-related precancerous conditions. In 2015, interim guidelines jointly issued by the American Society for Colposcopy and Cervical Pathology (ASCCP) and the Society of Gynecologic Oncology (SGO) recommended the incorporation of HPV testing as the primary screening for cervical cancer in women aged 25 and older [8]. Subsequently, in 2018, the U.S. Preventive Services Task Force (USPSTF) advised that women with an average risk of cervical cancer, aged 30–65, undergo HPV screening once every 5 years as a standalone method [9]. The global strategy for cervical cancer screening is progressively shifting from cytology-based approaches to HR-HPV screening. Despite this transition, the application of HPV testing in the context of cervical cancer screening during pregnancy has not been officially endorsed. The prevalence of HPV infection during pregnancy remains ambiguous, with reported rates varying widely, ranging from 9% to 35%, in different literature sources [10, 11]. Importantly, HPV infection during pregnancy can give rise not only to cervical lesions in pregnant women but also to complications such as genital condyloma acuminatum and laryngeal papilloma in the foetal anal genital area through vertical transmission from mother to foetus. Hence, the primary objective of this study was to assess the efficacy of the ThinPrep cytologic test (TCT) and the HPV DNA co-test in the realm of cervical cancer screening during pregnancy. This investigation aims to contribute valuable insights to the existing knowledge gap surrounding the optimal screening approaches for this specific population.

## Methods

### Study population and procedures

Between April 2016 and April 2019, this study conducted examinations of 8,712 pregnant women aged 21–49 years, all of whom had undergone prenatal visits at an obstetrics clinic without cervical cancer screening within the past year. The assessments included the HPV DNA test and the TCT administered during the 14th to 20th gestational weeks. Pregnant women with negative results in both HPV and cytology were advised to resume regular screening after a 3-year interval. Those presenting with HR-HPV positivity and/or cytologic abnormalities (specifically cytological diagnosis results for atypical squamous cells of undetermined significance or worse, ASCUS+) were promptly referred for colposcopy within 2–4 weeks following co-testing. Post-colposcopy, individuals suspected of harbouring a high-grade squamous intraepithelial lesion (HSIL) or cervical cancer underwent a cervical biopsy within 2 weeks, subsequent to comprehensive informed consent. Furthermore, pregnant women exhibiting HR-HPV positivity and/or cytologic abnormalities were scheduled for a follow-up 6 weeks postpartum. The primary end point for this evaluation was the detection of HSIL or lesions of a more severe nature.

TCTs were conducted using liquid-based cytology (ThinPrep Hologic). Abnormal cytology was defined as ASCUS+. The HPV tests were carried out utilising the 21 HPV GenoArray Diagnostic Kit (Hybribio Ltd.), which identifies 15 high‐risk types (HPV 16, 18, 31, 33, 35, 39, 45, 51, 52, 53, 56, 58, 59, 66, and 68) and 6 low-risk types (6, 11, 42, 43, 44, and 81). The methodology is grounded in the flow‐through hybridization principle.

### Histological classifications

Histological results were evaluated according to the 2014 WHO classification of female genital tumours. The histological classifications were as follows: normal, low‐grade squamous intraepithelial lesion (LSIL), HSIL, and squamous cell carcinoma (SCC).

### Statistical analysis

The collected data were analysed using SPSS software (version 16.0; SPSS, Inc.). Chi‐square and Fisher’s exact tests were utilized to evaluate the differences in the incidence of HSIL and SCC between the groups.

### Ethical considerations

All procedures performed in this study were conducted according to the ethical standards of the institutional and national research committee and the 1964 Declaration of Helsinki and its later amendments or comparable ethical standards. Informed consent was obtained from all included participants. The institutional review board of the Ren Ji Hospital, Shanghai Jiao Tong University School of Medicine approved this study. All pregnant women who performed screening signed informed consent, voluntarily accepted the cervical cancer screening programme during pregnancy, and agreed to its data collection and use.

## Results

### HPV infection rate and abnormal cytology rate during pregnancy

A total of 8,712 pregnant women were enrolled in this study, with an age range spanning from 21 to 49 years, with a mean age of 31 years and a median age of 29 years. Among these participants, 601 individuals were identified as harbouring HR-HPV, resulting in an HR-HPV infection rate of 6.90% (601/8712). Notably, pregnant women aged less than 25 years and those between 35 and 39 years exhibited elevated HR-HPV infection rates of 8.80% and 8.45%, respectively, surpassing other age groups significantly (*P* < 0.05; Table 1). Furthermore, 38 cases presented abnormal cytology results, yielding an abnormal cytology rate of 0.44% (38/8712). Of these cases, 18 were HR-HPV positive, including 14 cases of ASCUS, 3 cases of LSIL, and 1 case of atypical squamous cells that cannot exclude high-grade intraepithelial lesion (ASC-H). Conversely, 20 cases were HR-HPV negative, involving 3 cases of LSIL and 17 cases of ASCUS. Subsequently, 443 women with abnormal co-screening results underwent a colposcopy after providing comprehensive informed consent. The missing rate in this subset was 28.67% (178/621).

**Table 1.** Different age distribution of HPV infection

Age	Screening number	Infection number	Infection%
21–24	432	38	8.80^a^
25–29	3,448	219	6.35
30–34	3,170	210	6.62
35–39	1,385	117	8.45^a^
40~	277	17	6.14
Total	8,712	601	6.90

a The participants aged less than 25 years and those between 35 and 39 years exhibited elevated HR-HPV infection rates than other groups (*P* = 0.043).

### The HPV genotype and its relationship with the incidence of cervical lesions during pregnancy

Among the 601 cases identified with HR-HPV infection, 117 were associated with HPV 16/18, constituting 19.47% (117/601) of the total HR-HPV-positive cases (Table 2). The distribution of different types of HPV infections was then analyzed and ranked in descending order of frequency (Table 3). Notably, HPV 52 exhibited the highest positive rate, accounting for 18.47% of HR-HPV-positive patients, followed by HPV 16, 58, 51, and 53 in sequential order. Remarkably, while only one case of HPV 52 infection was diagnosed as HSIL, 11 cases of HPV 16 infection were associated with HSIL+, including two cases of SCC. This delineates a notable difference in the severity of the lesions associated with these HPV types.

**Table 2.** Proportion of the HPV16/18 and non-16/18 HPV infection cases

HPV genotype	*N*	% of total HR-HPV positive number (601)
16	74	12.31
18	18	3.00
16 and 18	1	0.17
16/18 + non-16/18	24	3.99
non-16/18	484	80.53
Total	601	100.00

**Table 3.** Proportion of HPV genotypes and its relationship with the HSIL+ and HPV16/18 (in 601 cases)

HPV genotype^a^	*N*	Biopsy number	HSIL+	Relative with HPV 16/18	% of total HR-HPV-positive number (601)
52	111	19	1	No	18.47
16	92	23	11(2 scc)	With1 case HPV 58 related	15.31
58	90	14	4	With1 case HPV 16 related	14.98
51	66	11	0	–	10.98
53	63	10	1	No	10.48
39	62	10	1	No	10.32
66	38	8	0	–	6.32
33	35	5	0	–	5.82
31	31	7	2	With1 case HPV 18 related	5.16
18	30	10	1	1 case with HPV 31	4.99
68	29	2	1	No	4.83
59	19	5	0	–	3.16
56	14	4	0	–	2.33
35	7	2	0	–	1.16
45	5	1	0	–	0.83

a The genotype count here counts how many cases the type appears in, so multiple infection patients are counted once per genotype.

Among the cohort of 601 patients with HR-HPV infection, 423 cases were referred for colposcopy, resulting in a referral rate of 70.38% (423/601). Out of these, 114 individuals with suspected HSIL+ underwent biopsies. Pathological examination revealed 19 cases with confirmed HSIL+ lesions, including 17 cases of HSIL and 2 cases of cervical cancer, indicating an incidence of 16.67% (19/114). Additionally, 60 cases exhibited normal pathology (52.63%, 60/114), and 35 cases showed pathological LSIL (30.70%, 35/114; Table 4). In the context of this study, the incidence of HSIL lesions during pregnancy was 195 per 100,000 (17/8712) and the incidence of cervical cancer was 23 per 100,000 (2/8712).

**Table 4.** Relationship between cervical biopsy histology and HPV typing in HR-HPV infection during pregnancy

HPV genotype	Biopsy number	Normal	LSIL	HSIL	SCC	(HSIL+SCC)% of biopsy number (114)	(HSIL+SCC)% of colposcopy number (443)	(HSIL+SCC)% of HR-HPV infection number (601)
16^a^	18	4	4	8	2			
18^b^	6	5	1	0	0			
16/18 + non-16/18^c^	9	3	4	2	0			
Subtotal	33	12	9	10	2	10.53%*	2.71%	2.00%
Non-16/18	81	48	26	7	0	6.14%	1.58%	1.16%
Total	114	60	35	17	2	16.67%	4.29%^**^	3.16%

HSIL: Including CIN II and CIN III. LSIL: CIN I. SCC: squamous cell carcinoma in cervix.

* HPV 16/18-related infection groups (a + b + c) and non-HPV 16/18 groups had significant differences in the percentage of lesions in HSIL and above, 10.53% vs. 6.14% (*P* < 0.05), and HPV16/18-related infection groups had higher rates of HSIL and above.

** The total patients referred to colposcopy included 423 HR-HPV-positive cases and 20 HR-HPV-negative ASCUS+ cases. The(HSIL+SCC)% of total cases referred to colposcopy was 4.29%.

Furthermore, an analysis was conducted of the relationship between HR-HPV genotypes and biopsy pathology indicative of precancerous lesions. Among the 19 patients with HSIL and above lesions, 10 with HSIL and 2 with cervical cancer were associated with HPV 16/18. In contrast, the remaining 7 patients with HSIL were linked to non-HPV 16/18 genotypes. The proportion of HPV 16/18-related HSIL+ lesions among the total HR-HPV-infected cases was 10.53%. In the non-HPV 16/18 group, HSIL+ lesions represented 6.14% of the total number of biopsies. The incidence of HSIL+ in the HPV 16/18-related group was significantly higher than in the non-HPV 16/18 group (*P* < 0.05; Table 4).

### Postpartum follow-up

The 256 patients with HR-HPV infection during pregnancy underwent TCT and HPV DNA co-screening again at 6 weeks postpartum. As shown in Table 5, 128 cases were HR-HPV negative and 128 cases were HR-HPV positive. Fourteen cases were reported as ASCUS, meaning that the cytology-positive rate was 5.47% (14/256), of which 5 cases were HPV negative. Among these 5 cases, 4 cases were examined by colposcopy and 1 case was diagnosed as LSIL.

**Table 5.** Postpartum follow-up of HPV infection

HPV infection status		*N*	% of follow-up patients (256)	% of HPV-positive patients (128)
HPV positive	Persistent infection	117	45.70	91.41
	Genotype change	11	4.30	8.59
HPV negative		128	50	–

Among the 128 HPV-negative patients after delivery, 33 cases had a biopsy during pregnancy, of which 2 cases were histologically confirmed HSIL; however, the biopsy pathologies after delivery of these 2 HSIL cases were negative. There were 11 cases of LSIL, and the postpartum co-testing showed that both the HPV test and TCT were negative; thus, they did not undergo a colposcopic-directed biopsy.

Among the 128 patients determined to be HPV positive after delivery, 117 cases remained the same HPV genotype (91.41%) and 11 cases changed the genotype of HR-HPV (8.59%). At 6 weeks postpartum, 70 patients underwent a colposcopic-directed biopsy. The pathological results showed 43 cases of normal (61.43%), 18 cases of LSIL (25.71%), and 9 cases of HSIL (12.86%; Table 6).

**Table 6.** Postpartum follow-up of pathologic diagnosis

Pathologic diagnosis	*N*	%
Normal	43	61.43
LSIL	18	25.71
HSIL	9	12.86
Total	70	

HSIL: Including CIN II and CIN III. LSIL: CIN I.

In the 128 patients determined to be HPV positive after delivery, 24 patients had a biopsy during pregnancy (normal in 6, LSIL in 9, and HSIL in 9). The histologic results were re-evaluated in 19 of 24 cases at 8–10 weeks postpartum. As shown in Table 7, a total of 9 cases showed a regression to normal or LSIL, 5 cases showed persistent LSIL, 3 cases had persistent HSIL, and 2 cases remained normal. The other 51 patients without a biopsy during pregnancy also had a colposcopic-directed biopsy at 8–10 weeks postpartum, and the histologic results suggested 33 cases of normal (64.71%, 33/51), 12 cases of LSIL (23.53%, 12/51), and 6 cases of HSIL (11.76%, 6/51; Table 7).

**Table 7.** The biopsy results during pregnancy and postpartum follow-up of 128 patients with HPV positive after delivery

Postpartum follow-up			Histology of biopsy during postpartum follow-up			
During Pregnancy			Normal	LSIL	HSIL	No colposcopy review
Histologic results	Normal	6	2	–	–	4
	LSIL	9	4	5	–	–
	HSIL	9	4	1	3	1
No histologic results		51	33	12	6	
Total		75	43	18	9	5

During pregnancy and postpartum, there were totally 28 cases with HSIL and above lesions. The HSIL+ detection rate was 4.66% (28/601) in the HR-HPV-positive co-screen patients.

### HSIL+ detection rates in different strategies for cervical cancer screening during pregnancy

Based on the current internationally approved cervical cancer screening strategies, this study compared HSIL+ detection rates using primary cytology screening, primary HPV screening, and co-testing, respectively. In strategy 1, which utilized cytology alone and a combination of HPV genotyping as a reflex test, patients with LSIL+ cytology or ASCUS HR-HPV-positive designations were referred for colposcopy. In strategy 2, which utilized primary HR-HPV screening with genotyping for HPV 16/18 and reflex cytology for patients with non-16/18 HR-HPV genotypes, patients with HPV 16/18-positive or non-16/18 HR-HPV-positive ASCUS+ designations were referred for colposcopy. In strategy 3, which utilized cytology and HR-HPV co-testing, patients with LSIL+ cytology or ASCUS non-16/18 HR-HPV-positive or HPV 16/18-positive designations were referred for colposcopy. As shown in Table 8, strategy 1 required the fewest referral colposcopies; however, it yielded the lowest HSIL+ detection rate and the highest number of missed HSIL+ among the three strategies. Furthermore, strategy 2 and strategy 3 had almost had the same HSIL+ detection rate and referral colposcopies.

**Table 8.** HSIL+ detection rate in different cervical cancer screening strategies during pregnancy

Screening strategy	Number of estimated colposcopies	Number of actual colposcopies	Detected HSIL+ number	Missed HSIL number	Number of colposcopies per HSIL+ detected	HSIL+ detection rate in 8712 (per 100,000)
Strategy 1	22	15	4	15	3.75	45.91
Strategy 2	130	99	13	6	7.62	149.22
Strategy 3	133	102	13	6	7.84	149.22

## Discussion

During pregnancy, the risk of cervical intraepithelial neoplasia (CIN) remains consistent with that of the non-pregnant period. Cervical screening holds paramount importance for pregnant women, and cervical cytology screening has been widely implemented in this demographic. The ASCCP guidelines recommend that pregnant patients undergo cytology testing at the initiation of their prenatal care. The incidence of cervical cytology abnormalities during pregnancy typically falls within the range of 1%–5%, with cytological abnormalities affecting 2%–7% of the 4 million maternal women in the United States annually [12]. Notably, a study in China reported that 8.12% of pregnant women exhibited abnormal cytology results at their first prenatal visit [13]. Contrary to these established norms, this study observed an unexpectedly low rate of cytological abnormalities during pregnancy. This anomaly could be attributed to factors such as bleeding during sampling, excessive vaginal mucous, and the inherent subjectivity of cytology [14]. In a prior retrospective analysis, the authors found that among HR-HPV-positive and TCT-negative non-pregnant patients, 15% displayed HSIL and above lesions [15]. Given the well-established higher sensitivity of HPV testing compared to cytology, surveillance with cytology alone is deemed acceptable in the general population only when HPV testing or co-testing is impractical [16]. However, it is noteworthy that, to date, HPV testing has not been incorporated into cervical cancer screening protocols for pregnant women.

HPV infection is prevalent among pregnant women, with the reported HR-HPV prevalence ranging from 9 to 35% during pregnancy [10]. In this study, the HR-HPV infection rate was 6.90% (601/8712), and notably, HPV 52 emerged as the most common genotype, diverging from the findings in other studies [17, 18, 19]. Nevertheless, these results align with previous research [20, 21], indicating that HPV 16/18 positivity poses the highest risk for HSIL and lesions of greater severity. This underscores the importance of heightened attention to pregnant women with HPV 16/18 infections compared to those with other HPV genotypes. Furthermore, this study’s comparison of HSIL detection rates using three screening strategies revealed that both primary HPV screening and co-testing exhibited higher sensitivity for detecting HSIL+ lesions. However, these strategies necessitated more colposcopy referrals in pregnant women than cytology alone. These findings suggest that HPV testing during pregnancy can effectively augment the relatively low sensitivity of cytology.

A prevailing challenge in cervical cancer screening in China is the insufficient coverage of screening programmes. Bao et al. investigated cervical cancer screening rates, considering both individual-level and geographical measures of socioeconomic status, and found that only 21.4% of 91,816 women aged ≥21 years had undergone cervical cancer screening [22]. For women who have never undergone cervical screening, the opportunity presented during pregnancy becomes pivotal for identifying potential issues. Consequently, despite the low risk of cervical cancer progression during pregnancy, this study advocates primary HPV screening and co-testing as effective screening strategies for pregnant women.

While pregnancy itself does not pose a heightened risk for the exacerbation of cervical lesions, it is imperative to conduct colposcopic surveillance in pregnant women with CIN to monitor for potential malignancy [23]. The prognosis of CIN during pregnancy has been a subject of varied outcomes in previous studies. Palle et al. analyzed the initial and postpartum histologies of 142 women, revealing that 25% exhibited spontaneous regression, 28% showed progression, and 47% displayed persistence. Additionally, two patients were diagnosed with microinvasive carcinoma in the postpartum period [24]. In a study by Vlahos et al. involving 78 pregnant women with CIN2+, the disease persisted in 30 patients (38.4%), while 48 patients (61.6%) experienced regression to CIN1 in the postpartum period [25]. Siddiqui et al. reported that a significant proportion of cervical dysplasia lesions demonstrated a high rate of regression (approximately 64%) and an extremely low rate of progression (approximately 3%) [26]. The current study’s 6-week postpartum follow-up revealed that 50% of HR-HPV infections during pregnancy were converted to negative. Among the 128 patients with persistent HPV positivity after delivery, 91.41% maintained the same HPV genotype, with 12.86% of these cases demonstrating HSIL upon cervical biopsy (Table 6). Notably, among patients with HSIL biopsy results during pregnancy, 37.5% exhibited persistent HSIL after delivery, which aligns with the findings of Vlahos et al. [25]. Additionally, the current study identified six cases of HSIL in 51 instances without biopsy during pregnancy (11.76%, 6/51; Table 7). Consequently, for patients with positive screening results during pregnancy, postpartum follow-up becomes indispensable.

In conclusion, this study systematically assessed the clinical efficacy of cytology alone, primary HPV screening, and co-testing for the detection of HSIL+ in pregnant women. The results demonstrated that HPV testing, either as a standalone method or in combination with cytology, exhibits feasibility and efficiency as a screening strategy for pregnant women. However, the implementation and evaluation of cervical cancer screening and follow-up during pregnancy present inherent challenges due to the unique characteristics of pregnant women. Positioned as a real-world research endeavour, this study contributes valuable insights into cervical cancer screening within the specific context of pregnancy.

## Data Availability

The data presented in this study are available upon request from the corresponding author. The data are not publicly available due to containing private information about individuals.
